# Light/dark phase influences intra-individual plasticity in maintenance metabolic rate and exploratory behavior independently in the Asiatic toad

**DOI:** 10.1186/s40850-022-00139-4

**Published:** 2022-07-11

**Authors:** Song Tan, Juan Li, Qiao Yang, Jinzhong Fu, Jingfeng Chen

**Affiliations:** 1 CAS Key Laboratory of Mountain Ecological Restoration and Bioresource Utilization & Ecological Restoration and Biodiversity Conservation Key Laboratory of Sichuan Province, Chengdu Institute of Biology, Chinese Academy of Sciences, Chengdu, 610041 China; 2 College of Life Sciences, Sichuan University, Chengdu, 610064 China; 3 University of Chinese Academy of Sciences, Shijingshan District, No.19 (A) Yuquan Road, Beijing, 100049 China; 4 Department of Integrative Biology, University of Guelph, Guelph, ON N1G 2W1 Canada

**Keywords:** light/dark phase, Exploratory behavior, Risk-taking behavior, Maintenance metabolic rate, Constraints, Amphibian

## Abstract

**Background:**

It is well-known that light/dark phase can affect energy expenditure and behaviors of most organisms; however, its influences on individuality (inter-individual variance) and plasticity (intra-individual variance), as well as their associations remain unclear. To approach this question, we repeatedly measured maintenance metabolic rate (MR), exploratory and risk-taking behaviors across light/dark phase four times using wild-caught female Asiatic toads (*Bufo gargarizans*), and partitioned their variance components with univariate and bivariate mixed-effects models.

**Results:**

The group means of maintenance MR and risk-taking behavior increased at night, while the group mean of exploratory behavior remained constant throughout the day. At night, the intra-individual variances were elevated in maintenance MR but reduced in exploration, suggesting that phenotypic plasticity was enhanced in the former but constrained in the latter. In addition, maintenance MR was not coupled with exploratory or risk-taking behaviors in daytime or at night, neither at the inter-individual nor intra-individual levels.

**Conclusions:**

Our findings suggest that these traits are independently modulated by the light/dark phase, and an allocation energy management model may be applicable in this species. This study sheds new insights into how amphibians adapt nocturnal lifestyle across multiple hierarchy levels via metabolic and behavioral adjustments.

**Supplementary Information:**

The online version contains supplementary material available at 10.1186/s40850-022-00139-4.

## Background

To cope with predictably rhythmic environments, organisms ranging from bacteria to plants and animals have developed internal timing mechanisms- biological clocks, enabling them to predict and prepare for changes in environmental conditions [1]. This internal timing system is governed by light, on both the daily (timing of light and dark) and seasonal (day length) scales. While there have been many comprehensive studies on how the light/dark phase affects the physiological and behavioral adjustment of different organisms, these studies have typically been restricted to comparing changes in group averages [2, 3]. However, as recent studies have shown, experimental biologists are increasingly interested in differences in behavior at the individual level, and relating them to differences in physiology [4–6]. Indeed, these studies often lead to fascinating insights into multiple ecological and evolutionary processes [7–9].

For most vertebrate species, willingness to explore and take risks is particularly interesting because these behavioral traits are closely related to resource acquisition and risk of predation [10–13]. Resource availability and predation risk periodically change between day and night, and an individual often accordingly adjusts its exploratory and risk-taking behavior, either independently or correlatedly (behavioral syndromes). As mentioned above, most prior works considered only the average response of exploration and risk-taking to the light/dark phase, and hence were lacking of variance partitioning [14–16], potentially resulting in inaccurate conclusions [2, 17, 18]. Therefore, characterizing the individuality (inter-individual variance, V_i_) and plasticity (intra-individual variance$,$ V_w_) of exploration and risk-taking across the light/dark phase may clarify and refine our understanding of the functional significance of these behaviors.

There are great interests among behavioral ecologists and ecophysiologists in exploring how metabolic physiology is associated with differences in behavior between individuals [5, 19–21]. Theoretically, maintenance metabolic rate (MR) (basal metabolic rate in endotherms, and routine metabolic rate or standard metabolic rate in ectotherms) can be used as a proxy for between-individual energy constraints. It can be positively, negatively, or not correlated with personality behaviors, depending on the energy-management models of the organism (i.e. how MR relates to total daily energy expenditure, DEE) [21, 22]. For instance, the performance model predicts that when the capacity of an organism to acquire energy is positively correlated with maintenance MR, behaviors that increase energy gain and expenditure should scale positively with maintenance MR [21–23]. A recent meta-analytical review revealed that the covariance patterns between maintenance MR and personality behaviors are consistent with the predictions of the performance model in a wide range of taxa, including birds, mammals, and reptiles [24, 25]. However, there are no such studies in amphibians, thus it is necessary to carefully examine the associations between energy metabolism and personality traits in this class to verify the generality of the models.

The light/dark phase may impact such association. The direction and strength of covariations between specific physiological and behavioral traits may be context-dependent and vary in relation to environmental conditions. It has been suggested that moderate stressors appear to reveal or amplify links between specific measures of physiology and behavior, whereas severe stressors might mask or attenuate any pre-existing relationships [26]. Unlike environmental stressors such as hypoxia [27], food deprivation [28], and extreme temperature [29], light/dark phase periodically regulates rather than challenge an individual’s homeostasis [30, 31]. If individuals vary in their physiological and behavioral sensitivity to the light/dark phase, the observed intraspecific phenotypic variation in these traits could either be coupled, or varied independently. Thus, considering the obvious ecological and evolutionary implications of trait integration and modularity [32–34], it is of great interest to examine how light/dark phase alters the relationship between maintenance MR and personality behaviors across hierarchal levels.

The Asiatic toad (*Bufo gargarizans*) provides a suitable study system to address this question, since field observations and laboratory quantitative analysis support that they are mostly, but not completely, nocturnal [35–38]. At high elevations, there is often a shift toward being more active in day time in toads [39]. In this study, we examined the effects of the light/dark phase on variability between and within individuals, as well as their covariations in Asiatic toads. We aimed to address the following questions: 1) Does the light/dark phase affect exploration and risk-taking behaviors, and maintenance MR? 2) Does the light–dark phase affect inter-individual and intra-individual variability of these traits? 3) Do exploration and risk-taking behaviors covary with maintenance MR across hierarchal levels (i.e. inter- and intra-individuals)? Are these covariations modulated by the light/dark phase? Because the nocturnal patterns are ancestral for this species and its close relatives, the fitness costs would be higher if they engage in activities (foraging, mate choice, dispersal etc.) during daytime than during nighttime [40–43]. Therefore, we predict that they would be more inclined to explore and take risks and increase maintenance metabolism at night in our experiments. Meanwhile, we hypothesize that the toads would exhibit greater plasticity (increase intra-individual variance) in maintenance MR and personality behaviors at night, because their nighttime activities are more unpredictable than their daytime ones [42, 43]. Finally, we predict that the associations between maintenance metabolic rate and personality traits would be strengthened at night, at both inter- and intra-individual levels, since this species faces stronger trade-offs in energy allocation at night [44, 45].

## Results

### Effects of the light–dark phase on trait averages

**Fig. 1:**
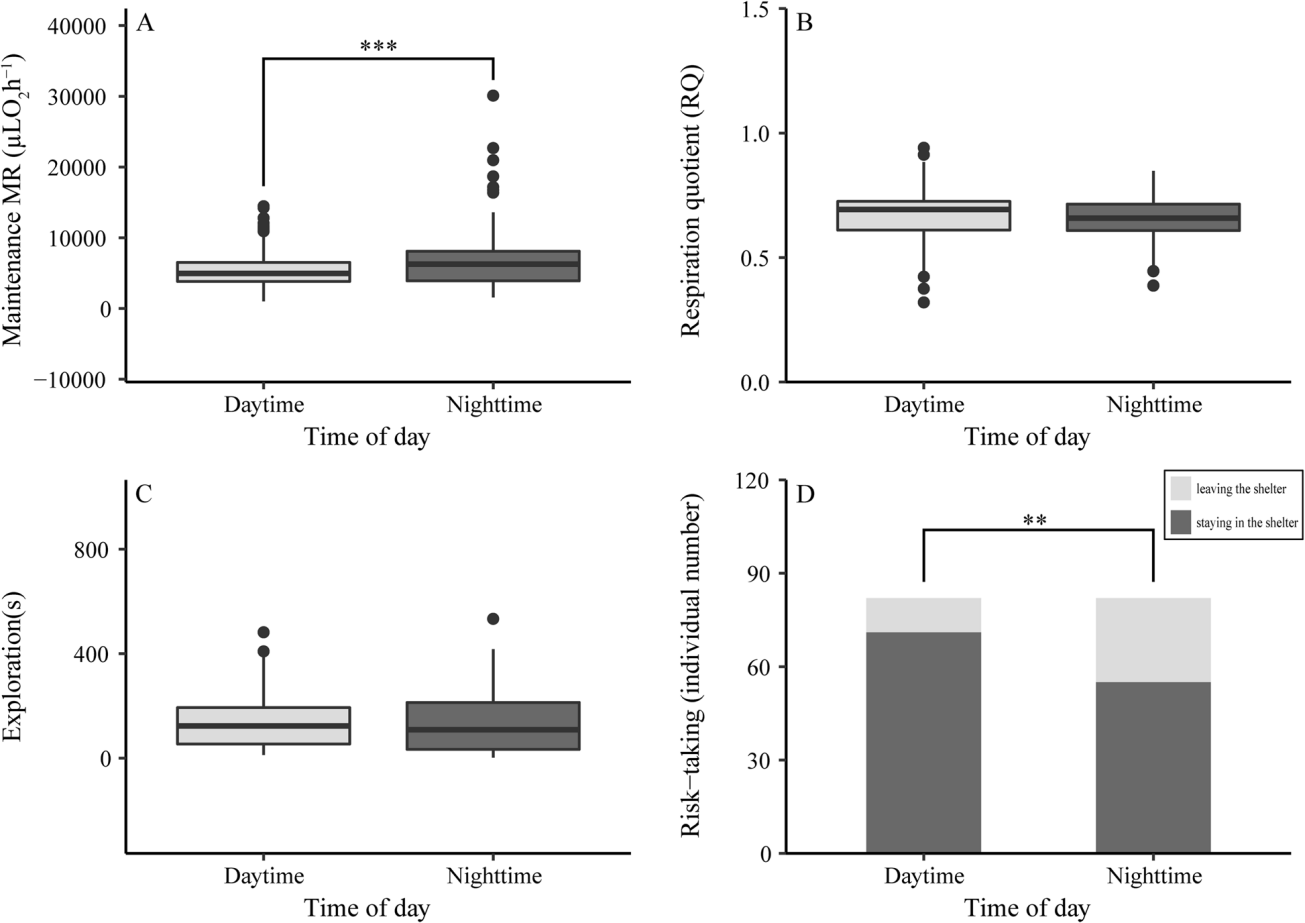
Summary results. **A**), estimated maintenance metabolic rate without fasting (MR_WF_: log-transformed); **B**), respiration quotient (RQ: exp-transformed); **C**), Exploration (Exploration: BOX-COX transformed) and **D**), risk-taking ("0" for coming out of the shelter and "1" for not coming out)*.* ** Statistically significant *P* value (*P* < 0.01). The box plots include five messages: “minimum” (Q1 minus 1.5*IQR), first quartile (Q1/25th percentile), median (Q2/50th percentile), third quartile (Q3/75th percentile) and "maximum" (Q3 plus 1.5 IQR), which are respectively represented by the nadir of the central line, the bottom line of the box, the middle line of the box, the top line of the box, and the peak point of the central line. Points above Q3 or under Q1 represent outliers. IQR is an abbreviation of interquartile range (the distance from the 25th to the 75th percentile)

Maintenance metabolic rate without fasting **(**MR_WF_) was significantly affected by the light–dark phase (*Pmcmc* < 0.001, Table 1), with higher oxygen consumption at night (Fig. 1A). In contrast, there were no detectable effects of the light/dark phase and trials on respiratory quotient (RQ) (*Pmcmc* > 0.05) (Table 1, Fig. 1B, Fig. S1). The light/dark phase only affected risk-taking behavior but not exploration willingness: the toads tended to be bolder at night (*Pmcmc* < 0.001) (Table 1, Fig. 1C, Fig. 1D). Moreover, we found that MR_WF_ showed a tendency to decrease across trials (*Pmcmc* < 0.05, Fig. S1) when body mass was controlled for (*Pmcmc* < 0.05) (Table 1, Fig. S2). The effects of body mass and trials on exploration and risk-taking traits were not significant (*P* > 0.05) (Table 1, Fig. S1).

**Table 1:** Fixed effects fitted to the univariate mixed model for scaled response variables maintenance metabolic rate without fasting (MR_WF_; uLO_2_ h^−1^), respiratory quotient (RQ), exploration (total move time) and risk-taking tendency (“yes” or “no”) in *Bufo gargarizans,* with fixed effects of light–dark cycle, body mass and Trials (trial number-effect of time). CI: credible interval; particle Markov chain Monte Carlo: pMCMC. pMCMC values < 0.01 are emphasized with bold

Trait	Posterior mean	Lower95% CI	Upper 95% CI	pMCMC
MR_WF_				
Intercept	-0.91	-1.88	-0.06	0.07
Light–dark cycle: nighttime	0.45	0.25	0.66	< 0.00
Body mass	0.01	0.00	0.01	0.05
Trials	-0.10	-0.19	-0.00	0.04
V_i_	0.64	0.27	1.14	NA
V_w_	0.41	0.32	0.54	NA
RQ				
Intercept	0.27	-0.20	0.71	0.24
Light–dark cycle: nighttime	-0.13	-0.38	0.14	0.31
Trial	-0.09	-0.21	0.03	0.14
V_i_	0.46	0.16	0.82	NA
V_w_	0.69	0.54	0.86	NA
Exploration				
Intercept	0.19	-0.70	1.05	0.66
Light–dark cycle: nighttime	-0.07	-0.34	0.19	0.60
Body mass	0.00	-0.01	0.01	0.96
Trials	-0.06	-0.19	0.06	0.34
V_i_	0.34	0.12	0.64	NA
V_w_	0.80	0.62	0.99	NA
Risk-taking				
Intercept	1.69	-1.08	4.41	0.22
Light/dark cycle: nighttime	-1.78	-2.91	-0.61	0.00
Body mass	0.01	-0.01	0.03	0.24
Trials	0.01	-0.51	0.48	0.97
V_i_	1.99	0.17	4.48	NA

### Effects of the light/dark phase on variance components

**Fig. 2:**
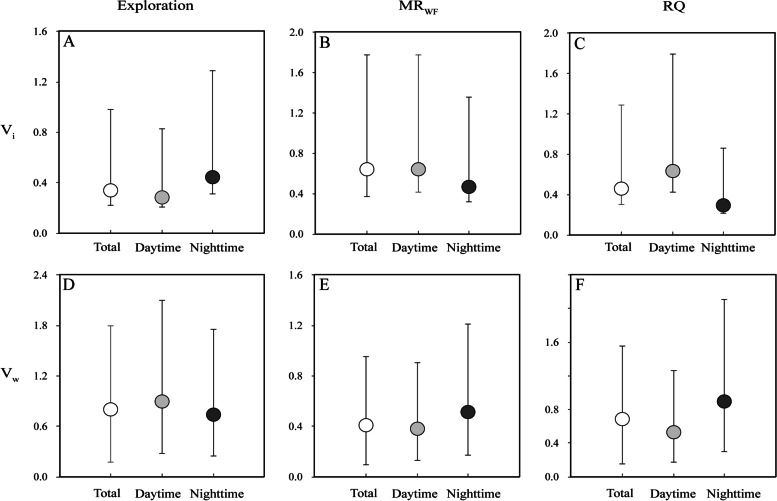
The between-individual variance (V_I_, A-C) and within-individual variances (V_e_, D-F) for exploration, maintenance metabolic rate without fasting (MR_WF_), and respiration quotient (RQ). Data are presented as mean ± 95% confidence interval

For MR_WF_, RQ, and exploration score, the model allowing differences at the intra-individual level (Model 3), and the model allowing differences at both the inter- and intra-individual levels (Model 4) had equivalent support (ΔDIC < 1.67, Table 2). The other two models were strongly rejected (Model 2 and Model 4, ΔDIC > 8, Table 2). These results indicated that the light/dark phase did not affect inter-individual variation but possibly affected intra-individual variation of these traits. However, the intra-individual variations of MR_WF_ and RQ at night were higher than those in the day (ΔV_w_, MR_WF_: -0.13 ± [-0.08, -0.17], RQ: -0.37 ± [-0.25, -0.47], Table S2, Fig. 2), while the opposite was seen for exploration (0.15 ± [0.12, 0.19], Table S2, Fig. 2). For the risk-taking score, the model allowing differences at the inter-individual levels was moderately supported (Model 2, ΔDIC = 4, Table 2), and the differences in the day were greater than those at night (1.24 ± [0.00, 5.42], Table S2).

**Table 2:** Model comparison for testing the effects of light–dark cycle on among-individual (V_i_) and within-individual variance (V_w_)

	Variance comparison	MR_WF_		RQ		Exploration		Risk-taking	
		DIC	ΔDIC	DIC	ΔDIC	DIC	ΔDIC	DIC	ΔDIC
Model 1	V_i_ = and V_w_ =	341.62	1.67	425.69	0.00	425.69	0.00	161.99	0.00
Model 2	V_i_ ≠ and V_w_ =	368.87	28.92	436.67	10.98	436.67	8.08	165.99	4.00
Model 3	V_i_ = and V_w_ ≠	339.95	0.00	426.7	1.02	426.71	1.35	NA	NA
Model 4	V_i_ ≠ and V_w_ ≠	367.31	27.36	436.37	10.68	436.37	9.90	NA	NA

### Effects of the light/dark phase on trait association

**Fig. 3:**
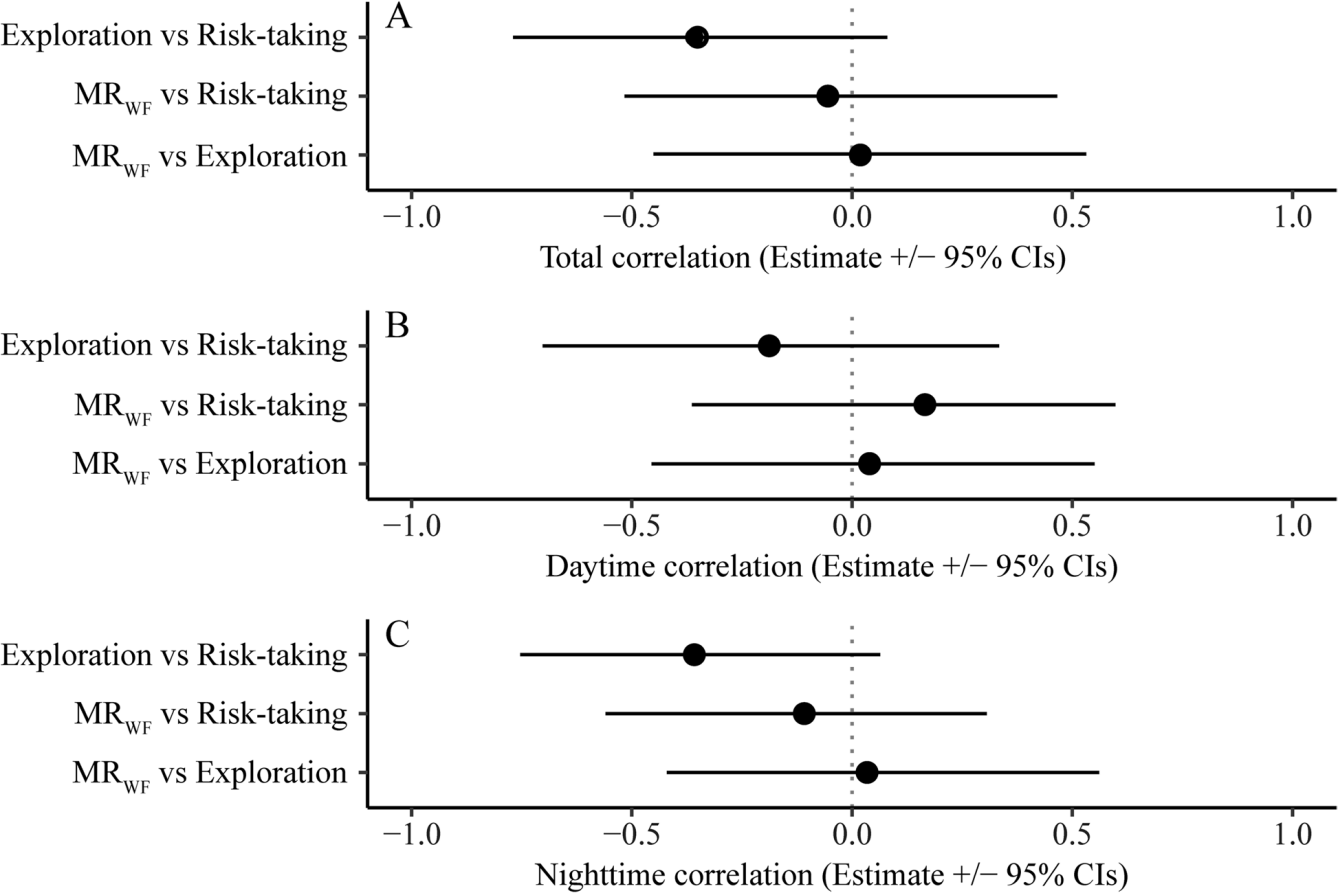
Estimated effect sizes for between-individual trait correlation (*r* ± 95% confidence interval) between maintenance metabolic rate without fasting (MR_WF_), exploration and risk-taking behavior. **A**, Total correlation; **B**, Daytime correlation; **C**, Nighttime correlation

We did not find significant covariances among MR_WF_, exploration, and risk-taking scores, at neither inter-individual nor intra-individual levels, during neither the day nor at night (Table S3, Fig. 3).

## Discussion

Asiatic toads clearly demonstrate increased metabolism and risk-taking tendency at night, but not exploratory behavior. Most animals in the wild display a diel variation in energy metabolism and behaviors. For example, energy expenditure usually increases during the photophase in diurnal species, and during or just before the scotophase in nocturnal species [46–49]. Consistent with our predictions, female Asiatic toads increased their MR_WF_ and the intensity of risk-taking at night. The elevated metabolic rate may assist them to counter reduced ambient temperature and maintain normal functionality such as food assimilation and immune defense at night [23, 50, 51]. Moreover, a greater willingness to take risks could motivate these nocturnal individuals to seek superior food resources or potential mates in limited time and space at night [52, 53]. Similar to risk-taking behaviors, evolutionary theory predicts that nocturnal species increase the intensity of exploratory behaviors to maximize food acquisition at night [54]. However, our results do not support this prediction. We observed a similar light/dark phase pattern of physical activity in our prior study as well [55]. One possible explanation is that their willingness to explore is dulled due to fully free-access to food; however, because exploration and risk-taking behaviors are usually intrinsically linked [13], this deduction is not particularly likely. An alternative explanation is that exploration and physical activities of Asiatic toads are constrained by the relatively small size of the test containers [56].

Asiatic toads increase metabolic flexibility during their active phase, while maintain a relatively constant metabolic level during the static phase. Our data clearly demonstrated that female toads increased the within-individual variance in MR_WF_ during the night. A major proportion of MR_WF_ is derived from food digestion, absorption, and assimilation in vertebrate [22]. Thus, the increase in metabolic flexibility may suit well with high variations in food processing during the nighttime. Generally, enhancing plasticity in exploration help individuals to cope with more unpredictable foraging environments and predation risk [57, 58]. For instance, when toads forage on habitat patches covered by abundant and homogeneously distributed food resources or clumped and ephemeral food sources, they may rapidly shift their searching strategies from thorough to superficial exploration. The degree of plasticity can have important fitness consequence [59] and it is well-established that animals can exhibit significant behavioral plasticity to both abiotic (e.g., temperature, diet quality, season changes) and biotic factors (predator, mate availability) [60–64]. Interestingly, contrary to our expectation, exploration willingness in Asiatic toads appears to be rather stable at night. One tentative explanation is that the environmental heterogeneity during the daytime is higher than that at night (food patches). For instance, aerial predators (e.g. birds) encountered mainly in the daytime are likely more unpredictable than ground predators (e.g. snakes) encountered mainly at night [65, 66].

Asiatic toads likely manage their energy budget in an allocation strategy, in which organisms work with a fixed energy budget and therefore daily energy expenditure (DEE) does not vary as a function of maintenance MR. Furthermore, variation in maintenance MR is not predicted to be associated with variation in behaviors that bring in net energy, but is predicted to be positively associated with energetically costly behaviors [21]. It is well known that risk-taking behaviors have consequences for net energy gains, but the expected relationship between maintenance MR and exploration will likely differ for different species or under different ecological contexts, depending on the intensity of exploration and the extent to which they determine the likelihood of encountering food [25, 50]. Compared to the high intensity exploration in small mammals (e.g., 16.8 min/hour in *Peromyscus maniculatus*) [11], female Asiatic toads explore their surroundings with relatively low intensity (5.6 min /hour). Furthermore, field observations also show that they are capable of obtaining enough foods via exploring a limited distance in non-breeding seasons (mean: 40.1 ± 1.5 m/day, minimum–maximum: 11.4–101.8 m/day) (T. Yang, unpublished radio telemetric data). These results suggest that exploratory behavior costs a small amount of energy and is more likely to be associated with large net energy gains. Therefore, the between-individual associations of MR_WF_ with exploration, as well as the phenotypic correlations (assumed to be identical to between-individual correlations [25]) of MR_WF_ with risk-taking in Asiatic toads are consistent with the predictions of the allocation energy management model [22]. Similar to our findings, no covariation between resting metabolic rate (RMR) and exploratory behavior was detected in wild-caught semiaquatic salamanders (*Desmognathus brimleyorum*) [67]. Thus, although the performance energy management model is popular in birds, mammals, and reptiles, the allocation model is likely more common in amphibians – a largely unexplored ectothermic group in terms of coadaptation between energetics and behaviors [25, 56, 68]. Among the above four vertebrate groups, amphibians indeed have the lowest energy systems, which potentially drive them to favor the allocation strategy that would maximize fitness by reducing maintenance costs [69].

Light/dark phase does not modulate the associations between behaviors and energy metabolism in Asiatic toads. Our results show that the associations between MR_WF_ and personality traits, as well as the relationships between personality traits, do not differ between the light/dark phase. Theoretically, the light/dark phase potentially affects trait covariation in two ways. One is mechanistic linkage, such as pleotropic genetic effects or a common physiological pathway that probably results from past selection on the developmental stability and homeostasis of an organism [70]. The other is correlational selection. The light–dark phase often influences the fitness outcomes among different combinations of metabolic and behavioral phenotypes, and consequently alters the intensity of their covariations [51]. However, similar associations across light/dark phase suggest that selection pressures on trait integrations between behaviors and metabolism during daytime or nighttime in Asiatic toads are similar.

It is notable that we only examined the covariations between maintenance MR and behaviors in females of *B. gargarizans*, and these associations could be sex-specific due to the sexual divergence of life-history and physiology [68, 71]. For example, inter-individual correlations in locomotor activity and RMR are either non-significant or negative in *Drosophila* females, but are consistently and significantly positive in males [53]. Our recent study also revealed sex-specific covariations between growth rate and physiological traits in Asiatic toads [72], suggesting a potential divergence in covariation structures of maintenance MR and behaviors between sexes. In addition, we did not include altitude as a co-variable in the present study due to limited sample size. Recent study has found that the toads from medium and high altitudes are more active than those from low altitudes during the daytime, but not during the nighttime [55], which suggests a genetic or development-mediated variations of intra- or inter- individual plasticity along altitudinal gradients. Future studies should explore these aspects.

## Conclusions

In summary, female Asiatic toads increase their phenotypic means of maintenance metabolic rate and risk-taking behavior at night, likely as an adaptive strategy to their nocturnal lifestyle. However, the light/dark phase modulates only intra-individual variability in personality traits and energy metabolism, with an increased variance in exploration and decreased variance in basal energy expenditure. In addition, maintenance MR and behaviors that bring in net energy (i.e. exploration and risk-taking) are not well integrated at night or in daytime, indicating an allocation energy management model in this species. Our study sheds new light on the evolution of personality traits and metabolic physiology in amphibians, a largely under-investigated group.

## Methods

### Animals

A total of 21 adult female Asiatic toads were collected from four sites along the Dadu River drainage in western China in April, 2019 (Suppl Table 1). All toads were transported to our laboratory at the Chengdu Institute of Biology, Chinese Academy of Sciences (CAS). In the laboratory, all toads were individually housed in plastic containers (35.5 × 25 × 15 cm, L × W × H), in which a piece of wet sponge (5 × 7 cm) and an ‘U’ -shaped tile (15 × 14 × 7 cm) were supplied in each container for water conservation and sheltering. All toads were acclimated at a constant setting (ambient temperature: 20 °C, 12 h: 12 h light–dark cycle) for one year according to our previous protocol [55]. The 12: 12 light–dark cycle is most commonly used in similar experimental design and an endogenous (circannual) rhythm of corticosterone likely plays an important role in the seasonal change of physiology the *Bufo* toads [73]. They were primarily fed with mealworms (*Tenebrio molitor*) dusted with calcium powder (EXO TERRA). To ensure the toads can eat fresh mealworms ad libitum, the food plates were cleaned and refilled with new mealworms every two days during acclimation. Crickets (*Gryllus rubens*) were also provided once a week. All animal procedures were carried out in accordance with approved protocols from the Animal Care and Use Committee at the Chengdu Institute of Biology, CAS (Permit number: 20180820) and the field collections were approved by Department of Forestry of Sichuan Province (Permit number: 20180710).

**Fig. 4:**
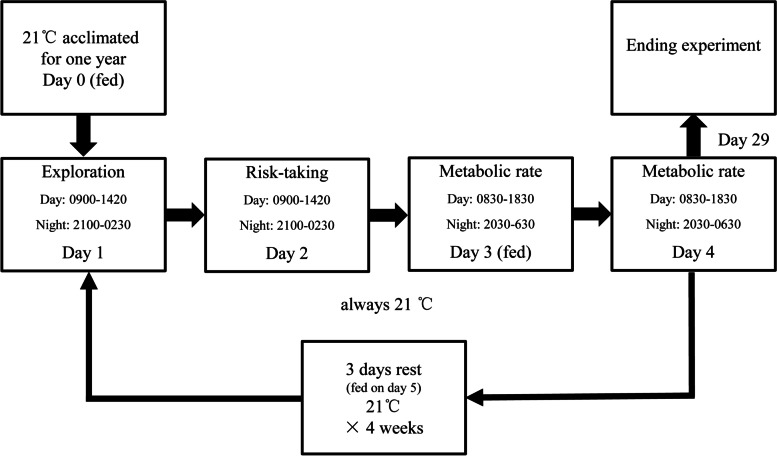
The time sequence of the experiments

We collected data on a 7-day schedule (Fig. 4). The first 2 days (days 1–2) were for behavioral observations, days 3 and 4 were for metabolic measurements, and followed by 3 days of resting. We repeated this 7-day sampling schedule for four consecutive weeks (four “bursts” of sampling). The feeding schedule during behavioral and metabolic measurements was the same as that during acclimation (Fig. 4). However, based on our observations, the toads ate rarely ate on day 3 (just before metabolic measurements), which was likely caused by hand stress manipulation.

### Behavioral assays

To quantify the personality traits in the toads, two separate behavioral assays were repeatedly conducted, examining behaviors along the exploration and risk-taking behavior axes. All toads experienced trial types in the same order, i.e., an exploratory trial in day 1 followed by a risk-taking trial in day 2. During each trial, we conducted the two behavioral assays twice, one between 0900 and 1430 h and one between 2100 and 0230 h. Therefore, each toad was subjected to 16 behavioral tests in total (8 for exploration and 8 for risk-taking). Trials took place in rectangular opaque plastic arenas (115 × 71 × 40 cm, L × W × H). The floor of each arena was fully covered with clean white paper, which was replaced between trials to eliminate scent cues. We also measured the temperature of arena substrate before the commencement of each trial (range: 20–21 °C). All trials were recorded using CCTV cameras (Boli, China) and the investigator left the room during trials to avoid interfering with the toad’s behavior. Behaviors were scored using video analysis and modelling tool “Tracker 5.2.0” (https://physlets.org/tracker/change_log.html).

**Fig. 5:**
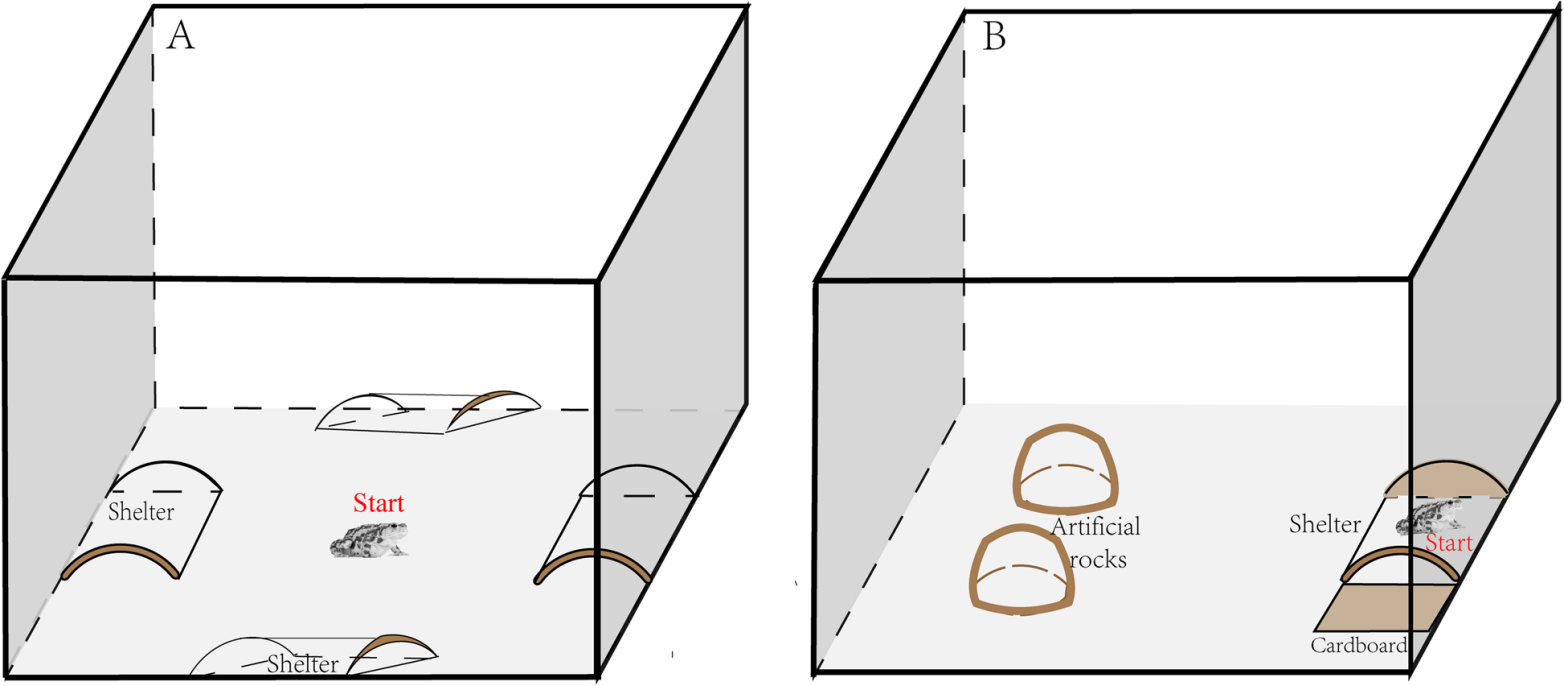
Test arenas used to examine personality behaviors in Asiatic toads (*Bufo gargarizans*). A, arena for exploration trials; B, arena for risk-taking trials

To assess exploratory behavior of the toads, a novel environment test was used to score an individual's 1) reaction to an unfamiliar environment and 2) willingness to explore [57]. The arena contained four symmetrically-spaced shelters (‘U’ -shaped tile), one along each side of the arena, allowing the option of seeking refuge (Fig. 5A). To begin a trial, we placed a toad under a rest shelter in the canter of the arena for 5 min. The rest shelter was then removed, and the toad was filmed for the next 25 min. We counted the total time of a toad moving (in seconds; as oppose to stationary) for the duration of the trial. Thus, high activity levels in this trial represent exploration, not shelter-seeking.

To assess risk-taking behavior of the toads, we conducted emergence behavior trials. The same test arenas were used, but with one shelter placed at one end of the arena and two artificial rocks placed at the opposite side of the arena to provide visual novelty [57] (Fig. 5B). The tile shelter was sealed with tapes at one side, and the other side (exit) was temporarily blocked with a paperboard. To begin a trial, we placed a toad in the shelter and allowed 5 min for the animal to settle down. We then gently lifted the paperboard cover allowing the option of leaving the shelter. Trials were filmed for 30 min. Our scores for risk-taking behavior were 1) whether a toad emerges from the shelter during a trial, and 2) the latency time to emerge (in seconds). We classified toads as having emerged from the shelter only when their entire body was visible. We allocated a score of 1800s to toads that did not emerge. Due to the low proportion of toads coming out of the shelters, risk-taking score was treated as a binary variable ("0" for coming out and "1" for not coming out).

### Maintenance metabolic rate

Our prior work has established that Asiatic toads take more than seven days to be postabsorptive due to their relatively large size [55]. To avoid the potential influence of prolonged fasting on exploration and risk-taking behaviors, we measured maintenance MR without fasting (MR_WF_) the day after behavioral tests using an automated flow-through respirometry system (TSE Systems, Germany) [55]. Metabolic chambers were housed in a temperature-controlled biochemical incubator set at 21 °C, which represents conditions ecologically relevant for the toads in the wild across an activity season [35]. We tested all toads in the same sequence and all measurements were taken between 0830 and 1830 h for daytime batches, and between 2030 and 0630 h for nighttime batches. Each batch included five toads, and each subject was measured for 5 h. In detail, each toad was firstly weighed (± 0.1 g) and then immediately transferred into a 900 mL cylindrical polypropylene chamber for measurements. Each chamber contained a small piece of moist facial tissue to prevent desiccation during the measurements. Source gas was pushed through Magnesium perchlorate (Cl_2_MgO_8_) columns prior to entering a mass flow controller (G246, TSE Systems, Germany), which regulated the flow rate through metabolic chambers. The flow rate was set at 200 mL/minute. The air stream exiting the chambers flowed into a gas switcher (G244, TSE Systems, Germany), which directed the air from a focal chamber through the gas analyzers. The effluent gas stream was sub-sampled in parallel through H_2_O scrubbers prior to entering an O_2_ (S104 [DOX], TSE Systems, Germany) and CO_2_ (5111-CO2, TSE Systems, Germany) analyzer. All subjects were removed from the chambers after measurement, weighed (± 0.1 g), and moved back to their original boxes. The data were averaged and collected every 1 s by a computer connected analogue to-digital converter (TSE Systems, Germany), and analyzed using a standard software (TSE Systems, Germany). MR_WF_ was calculated from the lowest rate of oxygen consumption over 5 min. We also calculated the respiratory quotient (RQ, the ratio of CO_2_ produced to O_2_ consumed) which allows inference about aerobic catabolism [74].

### Statistical analyses

To meet the assumptions of normality, MR_WF_ was log10-transformed, RQ was exp-transformed, and exploration scores were BOX-COX transformed. All continuous variables (body mass, MR_WF_, RQ, exploration score) were standardized to a mean of zero and a variance of one. All data analysis was conducted in R (version R4.0.3).

We first tested the effects of the light–dark phase and body mass on the average trait values of MR_WF_ and RQ, exploration and risk-taking scores using the MCMCglmm package [75] for Bayesian mixed models using Markov-chain Monte Carlo (MCMC) with 5 × 10^6^ iterations, 5 × 10^4^ burn-in period and a thinning interval of 50 iterations. Before running the MCMCglmm, an ‘uninformative’, parameter-expanded model prior (V = 1 and nu = 1.002) was specified, which was appropriate for trait error distributions (all traits were treated as “Gaussian”, except that risk-taking was “categorical”). All models included light–dark phase, body mass (except for RQ), and trials as fixed effects, and individual identity (ID) as a random effect. To ensure convergence and adequate chain mixing, five independent chains were executed and their posterior distributions and autocorrelation plots were compared.

Secondly, we tested the effects of the light–dark phase on trait variations at the between- and within individual levels. We separated our data into daytime and nighttime datasets, and compared the fit of four different univariate mixed models on all traits using MCMCglmm [60]. The models were as follows: Model 1- a null model where V_i_ and V_W_ were kept constant between daytime and nighttime datasets; Model 2—a model where V_i_ differed between datasets, while V_w_ was kept constant (V_i_ ≠ and V_w_ =); Model 3—a model where V_w_ differed between the daytime and nighttime datasets, while V_i_ was kept constant (V_i_ = and V_w_ ≠); Model 4—a model where both V_i_ and V_w_ were allowed to vary between the daytime and nighttime datasets. For risk-taking behavior, we tested only models 1 and 2 for the effects of the light/dark phase since V_w_ was fixed at value = 1 [75]. We then compared the deviance information criterion (DIC) among each model. The model with the lowest DIC values was considered the best model and those with ΔDIC > 5 were regarded as a significantly poorer fit. Models with ΔDIC < 5 were considered as having equivalent support compared to the best model. All models were specified with the same fixed effects structure as specified above to prevent biased estimates of variance components [60, 76]. The adjusted repeatability for MR, RQ, and exploration score were estimated by dividing their respective between-individual variance estimates by the sum of their between-individual and within-individual variances: (R_a_ = V_i_/ (V_i_ + V_w_)) [2, 77].

Lastly, we tested the effects of the light–dark phase on trait associations. We fitted bivariate mixed models to estimate the between- and within-individual covariances among MR_WF_, exploration and risk-taking scores using MCMCglmm. We used the same settings of iterations, burn-ins and thinning interval, as well as the same random and fixed effects, as the univariate models described above. Significance of the correlations associated with the light–dark phase was evaluated as above using 95% confidence intervals. We used a prior with V = diag (2) and nu = 1.002, assuming relatively uninformative for the correlations. However, we only analyzed inter-individual correlations for risk-taking behavior since it was a binary trait, and there was no residual variance for intra-individual analyses. We fixed the intra-individual variance for risk-taking behavior at 1.

## Supplementary Information

**Additional file 1.** 

**Additional file 2.** 

**Additional file 3.** 

**Additional file 4.** 

**Additional file 5.** 

## Data Availability

The dataset supporting the conclusions of this article is included within the article and its additional file.
