# Supplementary material for: Light/dark phase influences intra-individual plasticity in maintenance metabolic rate and exploratory behavior independently in the Asiatic toad
Source: BMC Zool. 2022 Jul 11;7:39. doi: 10.1186/s40850-022-00139-4 (PMC10127016; doi:10.1186/s40850-022-00139-4)
Supplement: Supplementary file 2 — Additional file 2. [file 40850_2022_139_MOESM2_ESM.pdf]

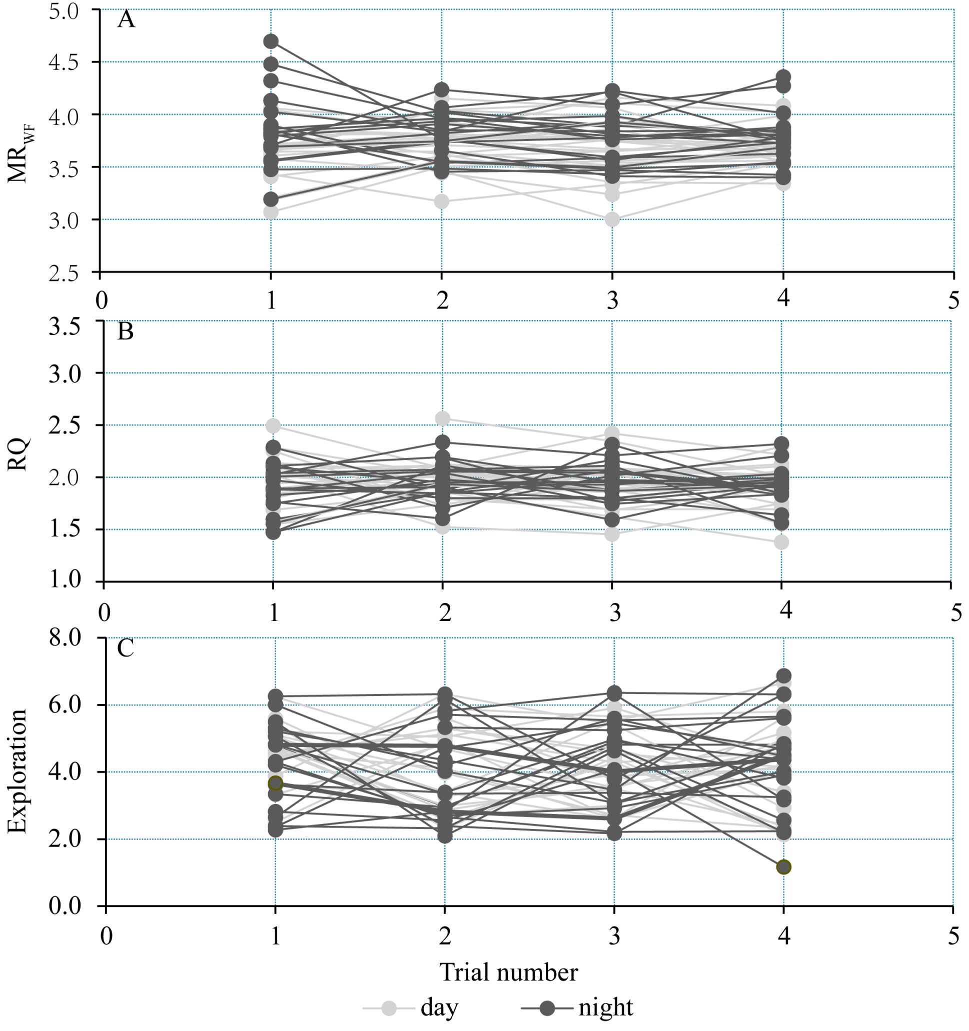

Fig S1 Trends of measurement values over the trial period. A, MR$_{WF}$; B, RQ; and C, Exploration (total moving time) along each trial in Asiatic toads (Bufo gargarizans) quantified in the light-dark cycle. Trials were separated by 3 days' rest.
