# Supplementary material for: Light/dark phase influences intra-individual plasticity in maintenance metabolic rate and exploratory behavior independently in the Asiatic toad
Source: BMC Zool. 2022 Jul 11;7:39. doi: 10.1186/s40850-022-00139-4 (PMC10127016; doi:10.1186/s40850-022-00139-4)
Supplement: Supplementary file 3 — Additional file 3. [file 40850_2022_139_MOESM3_ESM.pdf]

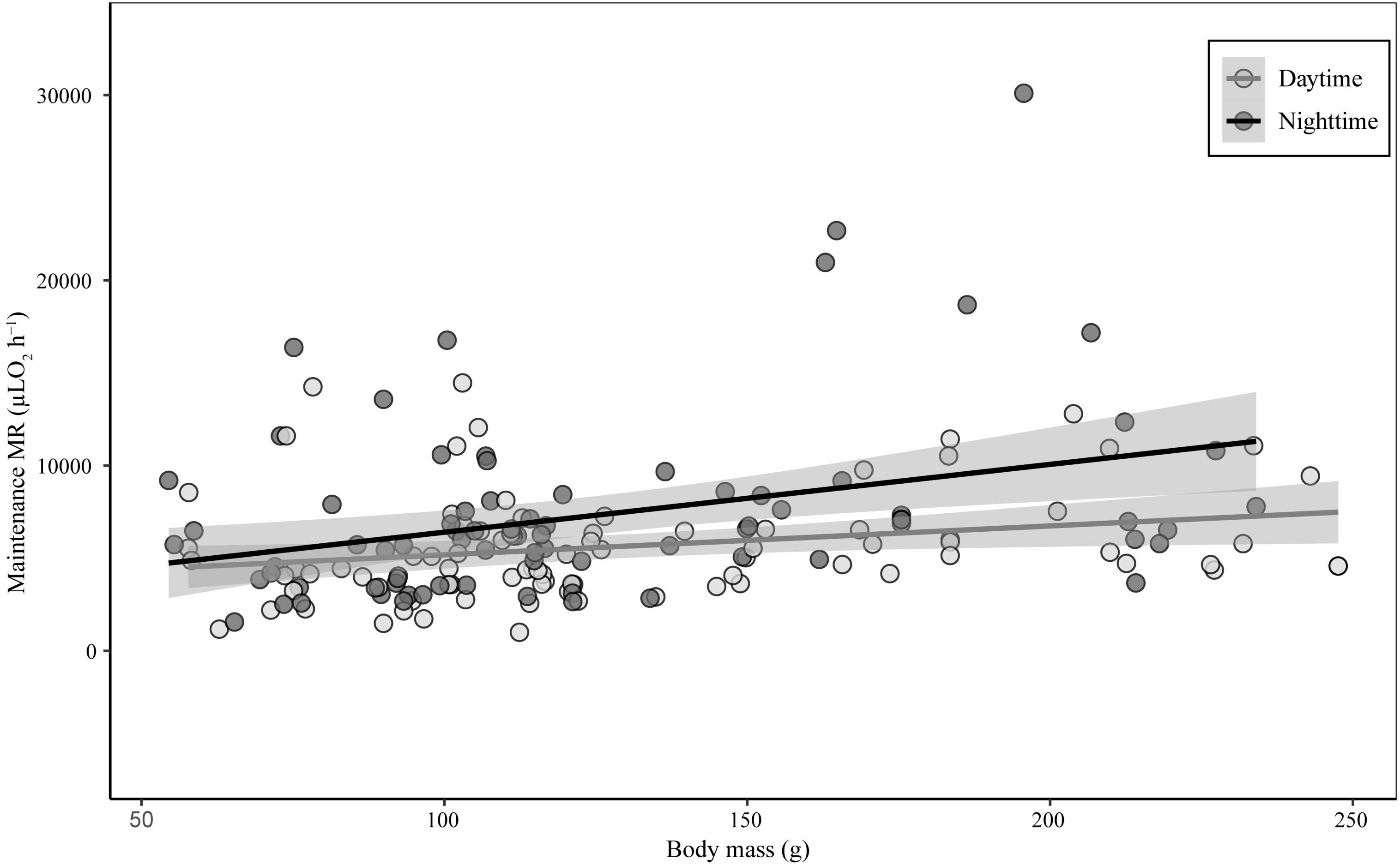

Fig S2 Maintenance metabolic rate (MR_{WF}, μL O_2·min^{-1}) as a function of body mass (mg) in Asiatic toads (Bufo gargarizans). MR_{WF} was log10-transformed.
