# Supplementary material for: Light/dark phase influences intra-individual plasticity in maintenance metabolic rate and exploratory behavior independently in the Asiatic toad
Source: BMC Zool. 2022 Jul 11;7:39. doi: 10.1186/s40850-022-00139-4 (PMC10127016; doi:10.1186/s40850-022-00139-4)
Supplement: Supplementary file 5 — Additional file 5. [file 40850_2022_139_MOESM5_ESM.docx]

Table S1 Collection sites of Asiatic toads (*Bufo gargarizans*) in Sichuan Province and their climatic characteristics

| Sites | Coordinates | Altitude (m) | Sample size | Mean annual temperature (℃)^*^ |
| --- | --- | --- | --- | --- |
| lingguan | 102°49′45.0″E, 30°14′58.5″N | 790 | 6 | 15.95 (22.6, 23.9) |
| dengchigou | 102°56′27.9″E, 30°32′50.6″N | 1590 | 5 | 16.49 (23.3, 24.6) |
| qiaoqi | 102°45′31.9″E, 30°39′44.5″N | 2010 | 6 | 5.99 (12.9, 13.8) |
| jiajinshan | 102°43′38.8″E, 30°48′19.2″N | 2500 | 4 | 3.85 (12.1, 13.4) |

*Climatic variables taken from WorldClim database (http://www.worldclim.org/).

#Values in parentheses includes mean temperature of the warmest quarter and mean of maximum temperature of the warmest month.

Table S2 Variance components (among-individual covariance, V_i_ ; within-individual variance, V_w_) and adjusted repeatability (R_a_) of maintenance metabolic rate (MR_WF_; uLO_2_ h^-1^), respiratory quotient (RQ), exploration (total move time), and risk-taking tendency (“yes” or “no”) in *Bufo gargarizans* across light-dark phase. Results are presented with posterior mode ±95% credible intervals.

| Trait | Total | Daytime | Nighttime | ΔV=V_Daytime -_V_Nighttime_ |
| --- | --- | --- | --- | --- |
| MR_WF_ |  |  |  |  |
| V_i_ | 0.64 (0.27, 1.14) | 0.64 (0.23, 1.14) | 0.47 (0.15, 0.89) | 0.17 (0.08, 0.25) |
| V_w_ | 0.41 (0.32, 0.54) | 0.38 (0.25, 0.53) | 0.51 (0.34, 0.70) | -0.13(-0.08, -0.18) |
| R_a_ | 0.60(0.41, 0.76) | 0.62(0.39, 0.80) | 0.43(0.25, 0.70) |  |
| RQ |  |  |  |  |
| V_i_ | 0.46 (0.16, 0.82) | 0.63 (0.20, 1.16) | 0.29 (0.07, 0.57) | 0.34(0.13, 0.60) |
| V_w_ | 0.69 (0.54, 0.86) | 0.53 (0.35, 0.74) | 0.90 (0.61, 1.21) | -0.37(-0.25, -0.47) |
| R_a_ | 0.37(0.20, 0.57) | 0.52(0.31, 0.74) | 0.21(0.08, 0.41) |  |
| Exploration |  |  |  |  |
| V_i_ | 0.34 (0.12, 0.64) | 0.28 (0.07, 0.55) | 0.44 (0.13, 0.85) | -0.16(-0.05, -0.3)0 |
| V_w_ | 0.80 (0.62, 0.99) | 0.89 (0.61, 1.21) | 0.74 (0.50, 1.02) | 0.15(0.12, 0.19) |
| R_a_ | 0.28(0.14, 0.46) | 0.17(0.08, 0.40) | 0.31(0.15, 0.57) |  |
| Risk-taking |  |  |  |  |
| V_i_ | 1.99 (0.17, 4.84) | 3.01 (0.09, 10.33) | 1.77 (0.09, 4.91) | 1.24(0.00, 5.42) |
| R_a_ | 0.50(0.13, 0.73) | 0.44(0.08, 0.86) | 0.41(0.76, 0.73) |  |

Table S3 Among-individual (*Covar _individual_*) and within-individual (*Covar _residual_*) correlations among maintenance metabolic rate without fasting (MR_WF_), exploration (total moving time), and risk-taking behavior in *Bufo gargarizans*. We present only the correlational data here. The complete results (including location effects and random effects) for each MCMCglmm can be found in Table S4. Results are presented with posterior mode ±95% credible intervals.

|  | Total | | Daytime | | Nighttime | |
| --- | --- | --- | --- | --- | --- | --- |
| Exploration *vs* Risk-taking | *Covar _individual_* | -0.57(-1.30, 0.04) | *Covar _individual_* | -0.39(-1.427, 0.389) | *Covar _individual_* | -0.44(-1.35, 0.23) |
| MR_WF_ *vs* Exploration | *Covar _individual_* | 0.06(-0.22, 0.39) | *Covar _individual_* | 0.08(-0.260, 0.408) | *Covar _individual_* | 0.01(-0.31, 0.36) |
|  | *Covar _residual_* | 0.09(-0.01, 0.19) | *Covar _residual_* | 0.02(-0.081, 0.249) | *Covar _residual_* | 0.13(-0.03, 0.31) |
| MR_WF_ *vs* Risk-taking | *Covar _individual_* | -0.10(-0.83, 0.62) | *Covar _individual_* | 0.21(-0.893, 1.369) | *Covar _individual_* | -0.16(-0.87, 0.52) |

Table S4 Estimates of fixed effects and variance components for resting metabolic rate (MR_WF_), exploration and risk-taking behavior in *Bufo gargarizans*, obtained from bivariate mixed models.

| Trait | Post.mean | l-95% CI | l-95% CI | pMCMC |
| --- | --- | --- | --- | --- |
| Total |  |  |  |  |
| MR_WF_ vs Total moving time |  |  |  |  |
| Fixed effects |  |  |  |  |
| MR_WF__O_2_ | -0.2660 | -0.6445 | 0.0997 | 0.1586 |
| Total moving time | 0.0427 | -0.2836 | 0.3604 | 0.8061 |
| MR_WF__O_2_: Trials | -0.0989 | -0.1860 | -0.0057 | 0.0337 |
| Total moving time: Trials | -0.0621 | -0.1887 | 0.0642 | 0.3275 |
| BM_1_ (MR_WF_) | 0.3356 | 0.0085 | 0.6588 | 0.0513 |
| BM_2_ (total moving time) | 0.0040 | -0.2679 | 0.2921 | 0.9721 |
| MR_WF_: Dark | 0.4546 | 0.2525 | 0.6439 | 0.0002 |
| Total moving time: Dark | -0.0734 | -0.3559 | 0.1887 | 0.5919 |
| Random effects |  |  |  |  |
| MR_WF_: MR_WF_ | 0.6766 | 0.2708 | 1.2272 |  |
| MR_WF_: Total moving time | 0.0616 | -0.2243 | 0.3868 |  |
| Total moving time: Total moving time | 0.3631 | 0.1284 | 0.6847 |  |
| Residual effects |  |  |  |  |
| MR_WF_: MR_WF_ | 0.4173 | 0.3263 | 0.5212 |  |
| MR_WF_: Total moving time | 0.0870 | -0.0076 | 0.1874 |  |
| Total moving time: Total moving time | 0.8032 | 0.6270 | 0.9990 |  |
| MR_WF_ vs Risk-taking |  |  |  |  |
| Fixed effects |  |  |  |  |
| MR_WF__O_2_ | -0.2530 | -0.6432 | 0.1099 | 0.1782 |
| Risk-taking | 2.6868 | 1.6864 | 3.7222 | <1e-04 |
| MR_WF__O_2_: Trials | -0.0878 | -0.2208 | 0.0592 | 0.2226 |
| Risk-taking: Trials | 0.0188 | -0.4373 | 0.4389 | 0.9305 |
| BM_1_ (MR_WF_) | 0.3681 | 0.0368 | 0.6949 | 0.3212 |
| BM_2_ (risk-taking) | 0.4277 | -0.3179 | 1.2223 | 0.2452 |
| MR_WF_: Dark | 0.4583 | 0.1474 | 0.7719 | 0.0049 |
| Risk-taking: Dark | -1.5952 | -2.6285 | -0.5946 | 0.0010 |
| Random effects |  |  |  |  |
| MR_WF_: MR_WF_ | 0.5360 | 0.1804 | 1.0230 |  |
| MR_WF_: Risk-taking | -0.1044 | -0.8299 | 0.6201 |  |
| Risk-taking: Risk-taking | 1.7813 | 0.1491 | 4.1971 |  |
| Total moving time vs Risk-taking |  |  |  |  |
| Fixed effects |  |  |  |  |
| Total moving time | 0.0430 | -0.2887 | 0.3790 | 0.7933 |
| Risk-taking | 2.7726 | 1.7111 | 3.9408 | <1e-04 |
| Total moving time: Trials | -0.0629 | -0.2017 | 0.0745 | 0.3729 |
| Risk-taking: Trials | 0.0371 | -0.4098 | 0.4757 | 0.8762 |
| BM_1_ (Total moving time) | 0.0217 | -0.2838 | 0.3143 | 0.8750 |
| BM_2_ (risk-taking) | 0.4630 | -0.3480 | 1.2908 | 0.2467 |
| Total moving time: Dark | -0.0741 | -0.3759 | 0.2325 | 0.6400 |
| Risk-taking: Dark | -1.6406 | -2.6444 | -0.5693 | 0.0016 |
| Random effects |  |  |  |  |
| Total moving time: Total moving time | 0.3883 | 0.1283 | 0.7248 |  |
| Total moving time: Risk-taking | -0.5657 | -1.2955 | 0.0348 |  |
| Risk-taking: Risk-taking | 2.2707 | 0.2374 | 5.2105 |  |
|  |  |  |  |  |
| Daytime |  |  |  |  |
| MR_WF_ vs Total moving time |  |  |  |  |
| Fixed effects |  |  |  |  |
| MR_WF__O_2_ | -0.0317 | -0.4161 | 0.3720 | 0.8760 |
| Total moving time | 0.0029 | -0.3184 | 0.3063 | 0.9784 |
| MR_WF__O_2_: Trials | -0.0687 | -0.2094 | 0.0582 | 0.3030 |
| Total moving time: Trials | -0.1505 | -0.3483 | 0.0268 | 0.1772 |
| BM_1_ (MR_WF_) | 0.3512 | -0.0191 | 0.7183 | 0.0636 |
| BM_2_ (total moving time) | 0.0562 | -0.2453 | 0.3664 | 0.7113 |
| Random effects |  |  |  |  |
| MR_WF_: MR_WF_ | 0.7469 | 0.2702 | 1.3968 |  |
| MR_WF_: Total moving time | 0.0779 | -0.2601 | 0.4082 |  |
| Total moving time: Total moving time | 0.2950 | 0.0841 | 0.5895 |  |
| Residual effects |  |  |  |  |
| MR_WF_: MR_WF_ | 0.4409 | 0.2866 | 0.6114 |  |
| MR_WF_: Total moving time | 0.0822 | -0.0809 | 0.2492 |  |
| Total moving time: Total moving time | 0.9112 | 0.6310 | 1.2441 |  |
| MR_WF_ vs Risk-taking |  |  |  |  |
| Fixed effects |  |  |  |  |
| MR_WF__O_2_ | -0.0130 | -0.4037 | 0.3653 | 0.9545 |
| Risk-taking | 2.8853 | 1.5780 | 4.4389 | <1e-04 |
| MR_WF__O_2_: Trials | -0.0513 | -0.2510 | 0.1439 | 0.6145 |
| Risk-taking: Trials | 0.0216 | -0.7222 | 0.7653 | 0.9570 |
| BM_1_ (MR_WF_) | 0.33056 | -0.0361 | 0.7181 | 0.0816 |
| BM_2_ (risk-taking) | 0.46771 | -0.7444 | 1.6257 | 0.3931 |
| Random effects |  |  |  |  |
| MR_WF_: MR_WF_ | 0.5537 | 0.1571 | 1.117 |  |
| MR_WF_: Risk-taking | 0.2143 | -0.8930 | 1.369 |  |
| Risk-taking: Risk-taking | 2.9905 | 0.0852 | 9.788 |  |
| Total moving time vs Risk-taking |  |  |  |  |
| Fixed effects |  |  |  |  |
| Total moving time | 0.0046 | -0.3185 | 0.3371 | 0.971 |
| Risk-taking | 3.0125 | 1.6247 | 4.6430 | <1e-04 |
| Total moving time: Trials | -0.1507 | -0.3387 | 0.0490 | 0.127 |
| Risk-taking: Trials | 0.0531 | -0.7242 | 0.8054 | 0.894 |
| BM_1_ (Total moving time) | 0.0569 | -0.2723 | 0.3547 | 0.720 |
| BM_2_ (risk-taking) | 0.5279 | -0.6608 | 1.7671 | 0.368 |
| Random effects |  |  |  |  |
| Total moving time: Total moving time | 0.3188 | 0.0810 | 0.6272 |  |
| Total moving time: Risk-taking | -0.3862 | -1.4271 | 0.3892 |  |
| Risk-taking: Risk-taking | 3.5578 | 0.1112 | 11.050 |  |
|  |  |  |  |  |
| Nighttime |  |  |  |  |
| MR_WF_ vs Total moving time |  |  |  |  |
| Fixed effects |  |  |  |  |
| MR_WF__O_2_ | -0.0215 | -0.3680 | 0.3065 | 0.8998 |
| Total moving time | 0.0035 | -0.3375 | 0.3411 | 0.9871 |
| MR_WF__O_2_: Trials | -0.1099 | -0.2558 | 0.0315 | 0.1366 |
| Total moving time: Trials | 0.0241 | -0.1519 | 0.1948 | 0.7879 |
| BM_1_ (MR_WF_) | 0.4198 | 0.0815 | 0.7345 | 0.0143 |
| BM_2_ (total moving time) | 0.0018 | -0.3204 | 0.3480 | 0.9889 |
| Random effects |  |  |  |  |
| MR_WF_: MR_WF_ | 0.4955 | 0.1668 | 0.9537 |  |
| MR_WF_: Total moving time | 0.006 | -0.3048 | 0.3167 |  |
| Total moving time: Total moving time | 0.4551 | 0.1271 | 0.8748 |  |
| Residual effects |  |  |  |  |
| MR_WF_: MR_WF_ | 0.5327 | 0.3492 | 0.7302 |  |
| MR_WF_: Total moving time | 0.1339 | -0.0252 | 0.3081 |  |
| Total moving time: Total moving time | 0.7586 | 0.5008 | 1.0363 |  |
| MR_WF_ vs Risk-taking |  |  |  |  |
| Fixed effects |  |  |  |  |
| MR_WF__O_2_ | -0.0152 | -0.3643 | 0.3287 | 0.9394 |
| Risk-taking | 1.0958 | 0.2532 | 1.9977 | 0.0097 |
| MR_WF__O_2_: Trials | -0.1006 | -0.3028 | 0.1061 | 0.3160 |
| Risk-taking: Trials | 0.0050 | -0.5452 | 0.5614 | 0.9846 |
| BM_1_ (MR_WF_) | 0.4247 | 0.0715 | 0.7518 | 0.0156 |
| BM_2_ (risk-taking) | 0.4979 | -0.3358 | 1.4403 | 0.2423 |
| Random effects |  |  |  |  |
| MR_WF_: MR_WF_ | 0.4089 | 0.1016 | 0.8300 |  |
| MR_WF_: Risk-taking | -0.1567 | -0.8738 | 0.5146 |  |
| Risk-taking: Risk-taking | 1.7304 | 0.1108 | 4.7277 |  |
| Total moving time vs Risk-taking |  |  |  |  |
| Fixed effects |  |  |  |  |
| Total moving time | 0.0040 | -0.3504 | 0.3852 | 0.9883 |
| Risk-taking | 1.0973 | 0.2315 | 2.0458 | 0.0125 |
| Total moving time: Trials | 0.0024 | -0.1695 | 0.2214 | 0.8077 |
| Risk-taking: Trials | 0.0083 | -0.5783 | 0.5386 | 0.9838 |
| BM_1_ (Total moving time) | 0.0051 | -0.3435 | 0.3695 | 0.9873 |
| BM_2_ (risk-taking) | 0.4719 | -0.4139 | 1.3792 | 0.2727 |
| Random effects |  |  |  |  |
| Total moving time: Total moving time | 0.4670 | 0.1224 | 0.9342 |  |
| Total moving time: Risk-taking | -0.4432 | -1.3529 | 0.2261 |  |
| Risk-taking: Risk-taking | 1.9876 | 0.1240 | 5.2200 |  |
